# Serum alpha-fetoprotein response as a preoperative prognostic indicator in unresectable hepatocellular carcinoma with salvage hepatectomy following conversion therapy: a multicenter retrospective study

**DOI:** 10.3389/fimmu.2024.1308543

**Published:** 2024-02-16

**Authors:** Kong-Ying Lin, Jian-Xi Zhang, Zhi-Wen Lin, Qing-Jing Chen, Liu-Ping Luo, Jin-Hong Chen, Kui Wang, Sheng Tai, Zhi-Bo Zhang, Shi-Feng Wang, Jing-Dong Li, Kai Wang, Lu Zheng, Si-Ming Zheng, Meng-Meng Wu, Ke-Can Lin, Tian Yang, Yong-Yi Zeng

**Affiliations:** ^1^ Department of Hepatopancreatobiliary Surgery, Mengchao Hepatobiliary Hospital of Fujian Medical University, Fuzhou, China; ^2^ Department of Hepatopancreatobiliary Surgery, First Affiliated Hospital of Fujian Medical University, Fuzhou, China; ^3^ Department of Hepatobiliary Surgery, Xiamen Hospital, Beijing University of Chinese Medicine, Xiamen, China; ^4^ Department of General Surgery, Huashan Hospital, Cancer Metastasis Institute, Fudan University, Shanghai, China; ^5^ Department of Hepatobiliary Surgery, Eastern Hepatobiliary Surgery Hospital, Navy Medical University (Second Military Medical University), Shanghai, China; ^6^ Department of Hepatopancreatobiliary Surgery, Second Affiliated Hospital of Harbin Medical University, Harbin, China; ^7^ Department of Hepatopancreatobiliary Surgery, Ganzhou Fifth People’s Hospital of Gannan Medical University, Ganzhou, China; ^8^ Department of Hepatopancreatobiliary Surgery, The Affiliated Hospital of North Sichuan Medical College, Sichuan, China; ^9^ Department of Hepatopancreatobiliary Surgery, Second Affiliated Hospital of Nanchang University, Nanchang, China; ^10^ Department of Hepatopancreatobiliary Surgery, Second Affiliated Hospital of Army Medical University, Chongqing, China; ^11^ Department of Hepatopancreatobiliary Surgery, First Affiliated Hospital of Ningbo University, Ningbo, China

**Keywords:** hepatocellular carcinoma, salvage resection, alpha-fetoprotein response, recurrence-free survival, conversion therapy

## Abstract

**Background:**

This study evaluates the efficacy of alpha-fetoprotein (AFP) response as a surrogate marker for determining recurrence-free survival (RFS) in patients with unresectable hepatocellular carcinoma (uHCC) who undergo salvage hepatectomy following conversion therapy with tyrosine kinase inhibitor (TKI) and anti-PD-1 antibody-based regimen.

**Methods:**

This multicenter retrospective study included 74 patients with uHCC and positive AFP (>20 ng/mL) at diagnosis, who underwent salvage hepatectomy after treatment with TKIs and anti-PD-1 antibody-based regimens. The association between AFP response—defined as a ≥ 80% decrease in final AFP levels before salvage hepatectomy from diagnosis—and RFS post-hepatectomy was investigated.

**Results:**

AFP responders demonstrated significantly better postoperative RFS compared to non-responders (*P*<0.001). The median RFS was not reached for AFP responders, with 1-year and 2-year RFS rates of 81.3% and 70.8%, respectively. In contrast, AFP non-responders had a median RFS of 7.43 months, with 1-year and 2-year RFS rates at 37.1% and 37.1%, respectively. Multivariate Cox regression analysis identified AFP response as an independent predictor of RFS. Integrating AFP response with radiologic tumor response facilitated further stratification of patients into distinct risk categories: those with radiologic remission experienced the most favorable RFS, followed by patients with partial response/stable disease and AFP response, and the least favorable RFS among patients with partial response/stable disease but without AFP response. Sensitivity analyses further confirmed the association between AFP response and improved RFS across various cutoff values and in patients with AFP ≥ 200 ng/mL at diagnosis (all *P*<0.05).

**Conclusion:**

The “20-80” rule based on AFP response could be helpful for clinicians to preoperatively stratify the risk of patients undergoing salvage hepatectomy, enabling identification and management of those unlikely to benefit from this procedure.

## Introduction

Liver resection constitutes the primary therapeutic modality for hepatocellular carcinoma (HCC) (1–4). Nevertheless, a considerable subset of patients is often precluded from liver resection in clinical settings, owing to extensive tumor burden, compromised liver function, or the presence of extrahepatic metastases. The emergence of multidisciplinary therapeutic strategies, anchored in surgical resection, offers potential curative avenues for those initially deemed inoperable for HCC (5–7). Numerous studies highlight the importance of salvage hepatectomy for patients who become eligible for surgical resection (8–10). Conceptually, Salvage hepatectomy has the potential to ensure total tumor removal, effectively circumventing the resistance and side effects associated with systemic or localized therapies, thereby enhancing long-term oncological outcomes. However, postoperative recurrence constitutes the principal hindrance to a favorable prognosis post-salvage hepatectomy (11, 12). Current clinical practice faces challenges due to the lack of preoperative criteria guiding salvage hepatectomy, such as identifying suitable patients or determining the optimal timing for the procedure (13). Given the invasive nature of salvage hepatectomy, there is a pressing necessity for dependable, replicable, and non-invasive preoperative prognostic markers that can accurately identify patients unlikely to benefit from this surgical approach.

Alpha-fetoprotein (AFP), a straightforward, rapid, and routinely employed clinical biomarker, is extensively utilized for HCC screening, diagnosis, and risk stratification (14–16). Serum AFP levels are recognized as an ideal surrogate biomarker for tumor burden, with higher levels often indicative of more aggressive tumor biology. The prognostic stratification capacity of AFP levels has been substantiated through numerous studies, leading to its incorporation as a pivotal variable in esteemed HCC prognostic scoring systems, such as the Cancer of the Liver Italian Program score (17), Chinese University Prognostic Index (18), Metroticket 2.0 model (15), and French AFP model (16). In addition to these roles, dynamic changes in AFP levels, commonly referred to as AFP response, have been validated as effective in monitoring the efficacy of anti-tumor treatments, where decreases post-treatment suggest an encouraging overall therapeutic response (19–21). Despite the lack of consensus in previous research regarding the precise definition of AFP response, with cutoff values often ranging between 20% and 50%, its utility as an alternative indicator for disease progression or reaction to various treatments—including radical resection, transarterial chemoembolization, tyrosine kinase inhibitors, immune checkpoint inhibitors, and combination therapies—has been widely acknowledged (22–25). The role of AFP response in risk stratification and treatment response evaluation continues to attract significant scientific interest.

In this multicenter, retrospective study, we enrolled 74 patients with initially unresectable HCC and positive AFP at diagnosis (> 20 ng/mL) who underwent salvage hepatectomy following treatment with a combination regimen of tyrosine kinase inhibitors (TKIs) and anti-PD-1 antibodies (α-PD-1). Our objective was to assess the utility of AFP response as a preoperative surrogate marker for recurrence-free survival after salvage hepatectomy. To our knowledge, this is the first study to explore the application of AFP response in patients undergoing salvage hepatectomy, potentially offering valuable insights for clinical decision-making in this context.

## Methods

### Study population

A retrospective analysis was performed on standardized data of 101 patients who underwent salvage hepatectomy following conversion therapy based on TKI and α-PD-1 for initially uHCC between November 25, 2019, and August 31, 2022, from 10 tertiary academic hospitals, including Mengchao Hepatobiliary Hospital of Fujian Medical University, Eastern Hepatobiliary Surgery Hospital of the Naval Medical University, Huashan Hospital Affiliated to Fudan University, First Affiliated Hospital of Fujian Medical University, Ganzhou Fifth People’s Hospital of Gannan Medical University, Second Affiliated Hospital of Harbin Medical University, Second Affiliated Hospital of Nanchang University, First Affiliated Hospital of Ningbo University, Second Affiliated Hospital of Army Medical University, and The Affiliated Hospital of North Sichuan Medical College. HCC diagnoses were based on the American Association for the Study of Liver Diseases guidelines and Chinese Guidelines for the Diagnosis and Treatment of Primary Liver Cancer (2, 3). Tumor unresectability was defined according to surgical technique limitations and/or oncological outcomes (13). Technical unresectability pertains to the inability to perform a safe radical resection (R0 resection), due to inadequate residual liver volume (<40% for cirrhotic patients and <30% for non-cirrhotic patients) or anatomical constraints associated with the tumor’s proximity to major vascular structures. Oncological unresectability involves advanced disease characterized by aggressive tumor features such as major vascular invasion, multiple lesions beyond the up-to-seven criteria, or extrahepatic metastases. The ethical committees of all involved medical centers approved this study, adhering to the Declaration of Helsinki guidelines. All patients provided written informed consent before initiating conversion therapy and undergoing salvage hepatectomy.

### Conventional regimens

As a retrospective observational study, no interventions were made to the patient’s treatment protocols. All conversion plans adhered to the latest evidence for effectiveness and safety in antitumor therapy for HCC, established through shared decision-making with the patients.

All patients received at least one cycle of TKI combined with α-PD-1 therapy, with almost all receiving concomitant transcatheter intra-arterial therapy including transarterial chemoembolization (TACE) or hepatic arterial infusion chemotherapy (HAIC). For patients who received transcatheter intra-arterial therapies, initiation of TKIs and α-PD-1 agents typically followed within one week post-treatment. The TKIs utilized in this study are all recommended by the Chinese primary liver cancer treatment guidelines for the first-line treatment of advanced HCC (lenvatinib and sorafenib), except for apatinib, which is recommended in combination with camrelizumab (α-PD-1) as the first-line treatment for advanced HCC by Chinese guidelines. The α-PD-1 agents in this study encompassed camrelizumab, tislelizumab, pembrolizumab, toripalimab, and sintilimab. The selection of each therapeutic agent was influenced by its availability, institutional preferences, and the financial circumstances of the patients. Administration of TKIs and α-PD-1 agents, including dosages, frequencies, and adjustments, adhered strictly to the guidelines specified on the respective drug labels.

Experienced interventional radiologists at each participating center performed both HAIC and TACE procedures. The HAIC protocol adhered to the FOLFOX-HAIC regimen, comprising Oxaliplatin (130 mg/m2) infused over 2-3 hours, followed by Leucovorin (400 mg/m2) over 1-2 hours, then a 5-Fluorouracil bolus (400 mg/m2), and a continuous infusion of 2400 mg/m2 over 23 hours (26). This procedure was repeated every three weeks, with each session involving temporary catheter insertion. For TACE, after achieving super-selective catheterization of the tumor’s arterial supply, a mixture of epirubicin and iodized oil (up to 30ml) was gradually introduced under fluoroscopic guidance. This continued until sufficient deposition of iodized oil in the tumor lesions and a noticeable reduction in the blood supply were achieved. This was followed by the injection of gelatin sponge particles, continuing until blood flow at the microcatheter tip ceased completely. The TACE procedure was repeated as deemed necessary.

### Salvage hepatectomy

During conversion therapy, patients’ general condition and tumor resectability were dynamically assessed. Eligibility for salvage hepatectomy was determined based on the following criteria: a) Feasibility of achieving a safe R0 resection; b) Maintenance of an adequate residual liver volume (≥30% for non-cirrhotic patients, ≥40% for cirrhotic patients); c) Sustained complete or partial response, or stable disease for a minimum of two months in intrahepatic lesions; d) Lack of continuous and severe adverse reactions during conversion therapy; e) Absence of explicit contraindications for liver resection. Prior to undergoing salvage hepatectomy, patients had tyrosine kinase inhibitors (TKIs) withdrawn at least one week preoperatively, and α-PD-1 inhibitors discontinued for a minimum of two weeks.

For complex hepatectomies, preoperative 3D reconstruction was routinely utilized to facilitate the development of a tailored surgical plan. This plan encompassed both anatomical and partial liver resections, determining the extent of resection based on factors such as tumor location, size, number, and the patient’s underlying liver condition. Laparoscopic or open hepatectomy was conducted as previously reported, with a preference for anatomical liver resection where applicable. Extent of resection is divided into major hepatectomy and minor hepatectomy. Major hepatectomy is defined as the resection of 3 or more Couinaud segments, while minor hepatectomy is defined as the resection of less than 3 Couinaud segments (27).

### Definition of AFP response

Data on AFP levels at the time of diagnosis and at the final assessment were collected, each measured within one week prior to conversion therapy and salvage hepatectomy, respectively. The formula for calculating AFP variation (%) is defined as: (AFP _at diagnosis_ - AFP _final_)/AFP _at diagnosis_ *100 (%). The optimal cutoff for 80% AFP variation was established using Xtile software, with recurrence-free survival following salvage hepatectomy as the endpoint event (28). Based on this threshold, patients were categorized into two groups: AFP responders (AFP variation ≥80%) and non-responders (AFP variation <80%). To minimize cohort heterogeneity, sensitivity analysis was conducted using previously published AFP response cutoffs of 50% or 20%, to comprehensively assess the clinical relevance of AFP response in predicting recurrence-free survival following salvage hepatectomy (20, 21). Additionally, given the potential for non-tumor-related AFP elevations or fluctuations, particularly in patients with concomitant cirrhosis or hepatitis (typically not exceeding 200ng/mL), a further sensitivity analysis was performed on a subgroup with baseline AFP levels ≥200ng/mL (21, 29, 30).

### Postoperative follow-up

After discharge, patients were monitored in outpatient clinics using a standardized surveillance protocol for HCC recurrence. Follow-up visits were scheduled every 2-3 months during the first 2 years post-hepatectomy, followed by biannual visits thereafter. This follow-up regimen encompassed liver function assessments, serum AFP testing, chest computed tomography (CT) scans, abdominal ultrasounds, and liver contrast-enhanced CT or magnetic resonance imaging. Additional diagnostic procedures, such as bone scans or positron emission tomography scans, were employed as indicated for patients with suspected recurrence.

The primary endpoint of this study was recurrence-free survival (RFS) after salvage hepatectomy, which was defined as the time interval from the date of salvage hepatectomy to tumor recurrence, death, or the last follow-up, whichever occurred earliest.

### Statistics

Descriptive statistics were used to analyze the demographic and clinical data of patients. Continuous variables were expressed as mean ± standard deviation or median (interquartile range), and differences were tested using Student’s *t*-test or the Mann-Whitney *U*-test. Categorical variables were expressed as frequencies (percentages) and tested using chi-square tests or Fisher’s exact tests. RFS after salvage hepatectomy was depicted using the Kaplan-Meier method, and differences between groups were compared using the log-rank test. A multivariable Cox proportional hazards model was used to assess the preoperative prognostic value of AFP response, adjusting for potential confounding clinical variables. Preoperative clinical variables with a *P* value less than 0.1 in univariate analysis were included in the multivariable Cox proportional hazards regression analysis to determine the final independent preoperative risk factors. All statistical analyses were two-tailed, and differences with a P value less than 0.05 were considered statistically significant. Statistical analyses were performed using SPSS version 20 (SPSS, Inc., Chicago, IL, USA) and R version 4.1.1 (R Project, Vienna, Austria).

## Results

### Patient characteristics, conversion regimens, and salvage hepatectomy

**Figure 1:**
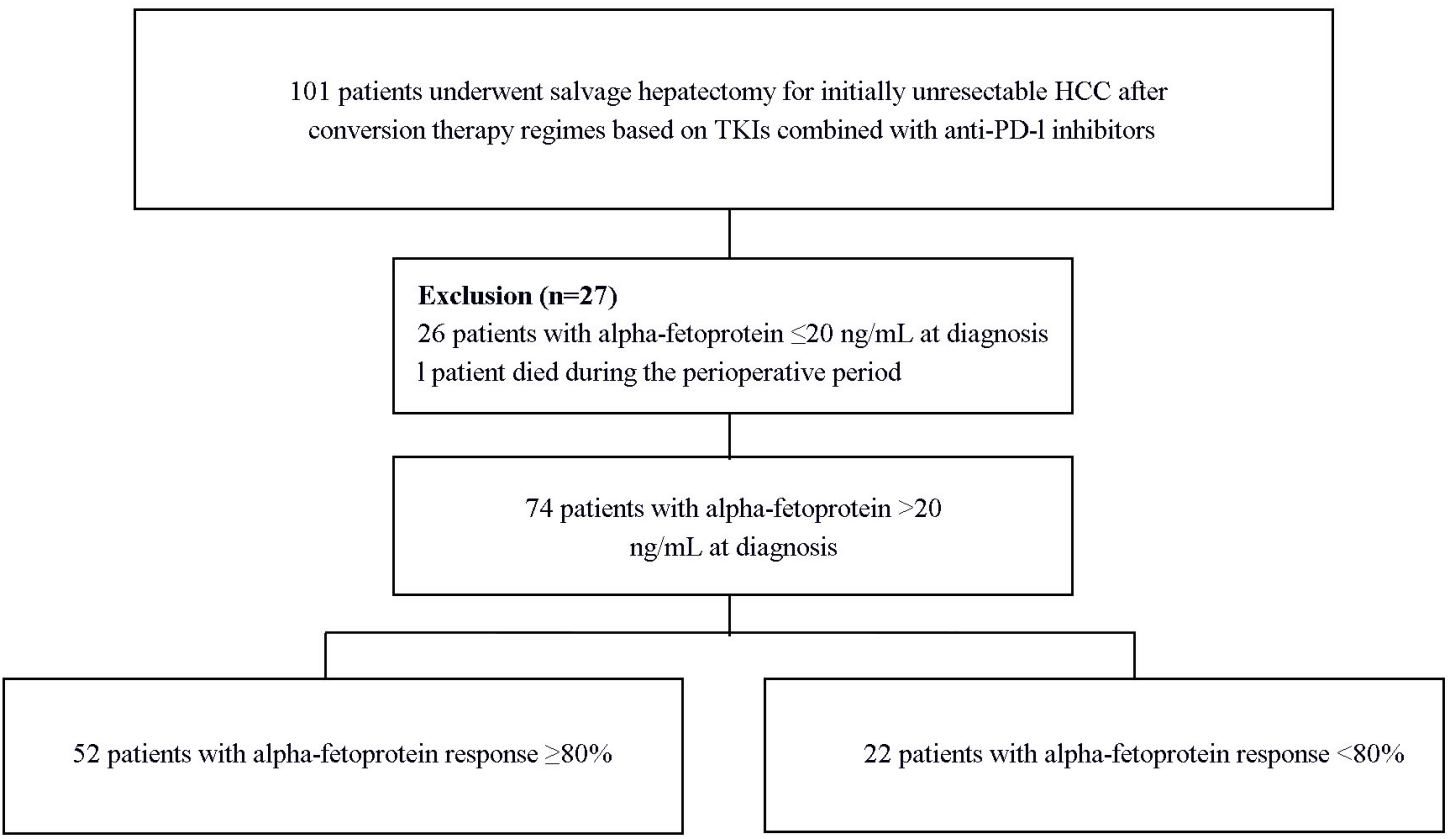
Selection of the study population. HCC hepatocellular carcinoma, PD-1, programmed death 1.

A total of 74 patients with positive serum AFP levels (defined as > 20 ng/mL) at diagnosis were included in the study (**Figure 1**). The mean age was 52.6 ± 12.3 years, with the majority being male (60, 81.1%). Hepatitis B virus infection was the predominant etiology (65, 87.8%). Liver cirrhosis was present in 44 (59.5%) patients, and 70 (94.6%) were classified as Child-Pugh A. Regarding tumor characteristics, 34 (45.9%) patients had a single lesion, the mean tumor size was 9.83 ± 4.36 cm, and 58 (78.4%) presented with macrovascular invasion (**Table 1**).

**Table 1:** Patient baseline characteristics.

Characteristics	Total cohort (n = 74)	AFP response, <80% (n = 22)	AFP response, ≥80% (n = 52)	*P Value*
**Age**, years, Mean (SD)	52.6 (12.3)	49.1 (10.9)	54.0 (12.6)	0.096
**Gender**, Male/Female	60 (81.1%)/14 (18.9%)	15 (68.2%)/7 (31.8%)	45 (86.5%)/7 (13.5%)	0.103
**Etiology**, HBV/HCV/Non-viral	65 (87.8%)/1 (1.4%)/8 (10.8%)	18 (81.8%)/0 (0%)/4 (18.2%)	47 (90.4%)/1 (1.9%)/4 (7.7%)	0.459
**Child-Pugh class**, A/B	70 (94.6%)/4 (5.4%)	20 (90.9%)/2 (9.1%)	50 (96.2%)/2 (3.8%)	0.577
**Liver cirrhosis**, Present/Absent	44 (59.5%)/30 (40.5%)	12 (54.5%)/10 (45.5%)	32 (61.5%)/20 (38.5%)	0.575
**TKIs,** Sorafenib/Lenvatinib/Apatinib	15 (20.3%)/51 (68.9%)/8 (10.8%)	3 (13.6%)/15 (68.2%)/4 (18.2%)	12 (23.1%)/36 (69.2%)/4 (7.7%)	0.379
**α-PD-1,** Camrelizumab/Tislelizumab/Toripalimab/Sintilimab/Pembrolizumab	35 (47.3%)/6 (8.1%)/4 (5.4%)/16 (21.6%)/13 (17.6%)	11 (50.0%)/3 (13.6%)/1 (4.5%)/5 (22.7%)/2 (9.1%)	24 (46.2%)/3 (5.8%)/3 (5.8%)/11 (21.2%)/11 (21.2%)	0.627
**Combine TACE**	61 (82.4%)	19 (86.4%)	42 (80.8%)	0.742
**Combine HAIC**	12 (16.2%)	3 (13.6%)	9 (17.3%)	1.000
**Conversion time**, Median (IQR), months	3.44 (2.44, 5.55)	3.28 (2.23, 6.08)	3.44 (2.57, 5.25)	0.799
**Radiological response,** CR/PR/SD	15 (20.3%)/45 (60.8%)/14 (18.9%)	2 (9.1%)/14 (63.6%)/6 (27.3%)	13 (25.0%)/31 (59.6%)/8 (15.4%)	0.233
AFP at diagnosis, ng/ml				
Median (IQR), ng/ml	1210 (292, 8620)	1210 (159, 2460)	1770 (561, 22900)	**0.016**
<1000/≥1000, ng/ml	31 (41.9%)/43 (58.1%)	10 (45.5%)/12 (54.5%)	21 (40.4%)/31 (59.6%)	0.686
Final AFP, ng/ml				
Median (IQR), ng/ml	44.9 (7.78, 835)	1040 (110, 4030)	13.2 (6.31, 115)	0.081
<1000/≥1000, ng/ml	57 (77.0%)/17 (23.0%)	11 (50.0%)/11 (50.0%)	46 (88.5%)/6 (11.5%)	**<0.001**
**Operative time**, Median (IQR), minutes	215 (184, 258)	223 (182, 281)	214 (189, 245)	0.420
**Surgical type**, Open/Laparotomy	54 (73.0%)/20 (27.0%)	15 (68.2%)/7 (31.8%)	39 (75.0%)/13 (25.0%)	0.546
**Intraoperative blood loss**, Median (IQR), ml	325 (200, 800)	375 (200, 800)	325 (200, 625)	0.962
**Intraoperative blood transfusion**, Yes/No	21 (28.4%)/53 (71.6%)	8 (36.4%)/14 (63.6%)	13 (25.0%)/39 (75.0%)	0.322
**Extend of hepatectomy**, Major/Minor	49 (66.2%)/25 (33.8%)	15 (68.2%)/7 (31.8%)	34 (65.4%)/18 (34.6%)	0.816
**Postoperative hospital day**, Median, (IQR), days	11.0 (9.00, 14.0)	10.5 (9.25, 13.8)	11.0 (8.75, 14.0)	0.206
**Tumor number**, Solitary/Multiple	34 (45.9%)/40 (54.1%)	11 (50.0%)/11 (50.0%)	23 (44.2%)/29 (55.8%)	0.649
**Tumor diameter**, Mean (SD), cm	9.83 (4.36)	10.3 (4.73)	9.64 (4.23)	0.600
**Macrovascular invasion**, Present/Absent	58 (78.4%)/16 (21.6%)	15 (68.2%)/7 (31.8%)	43 (82.7%)/9 (17.3%)	0.218
**MVI**, Present/Absent	21 (28.4%)/53 (71.6%)	8 (36.4%)/14 (63.6%)	13 (25.0%)/39 (75.0%)	0.322
**Pathological complete response**, Yes/No	18 (24.3%)/56 (75.7%)	1 (4.5%)/21 (95.5%)	17 (32.7%)/35 (67.3%)	**0.009**
**BCLC staging system**, A/B/C	4 (5.4%)/12 (16.2%)/58 (78.4%)	2 (9.1%)/5 (22.7%)/15 (68.2%)	2 (3.8%)/7 (13.5%)/43 (82.7%)	0.299

HBV, hepatitis B virus; HCV, hepatitis C virus; TACE, transarterial chemoembolization; HAIC, hepatic arterial infusion chemotherapy; TKI, tyrosine Kinase Inhibitor; PD-1, programmed death 1; AFP, alpha-fetoprotein; CR, complete response; PR, partial response; SD, stable disease; MVI, microvascular invasion; BCLC, Barcelona Clinic Liver Cancer; SD, standard deviation; IQR interquartile range.

Bold indicates statistical significance.

Almost all patients (73, 98.6%) received conversion therapy comprising TKI combined with α-PD-1 and concomitant transcatheter intra-arterial therapy. The median conversion time was 3.44 months (interquartile range [IQR], 2.44, 5.55). Radiological tumor response, assessed using the modified Response Evaluation Criteria in Solid Tumors, showed that 15 (20.3%) patients achieved complete response, 45 (60.8%) achieved partial response, and 14 (18.9%) had stable disease. The primary surgical approaches for salvage hepatectomy were open surgery (54, 73.0%), and most patients (49, 66.2%) underwent major liver resection. Postoperative pathological evaluation revealed that 18 patients (24.3%) achieved a pathological complete response (**Table 1**).

### AFP variations and AFP response

**Figure 2:**
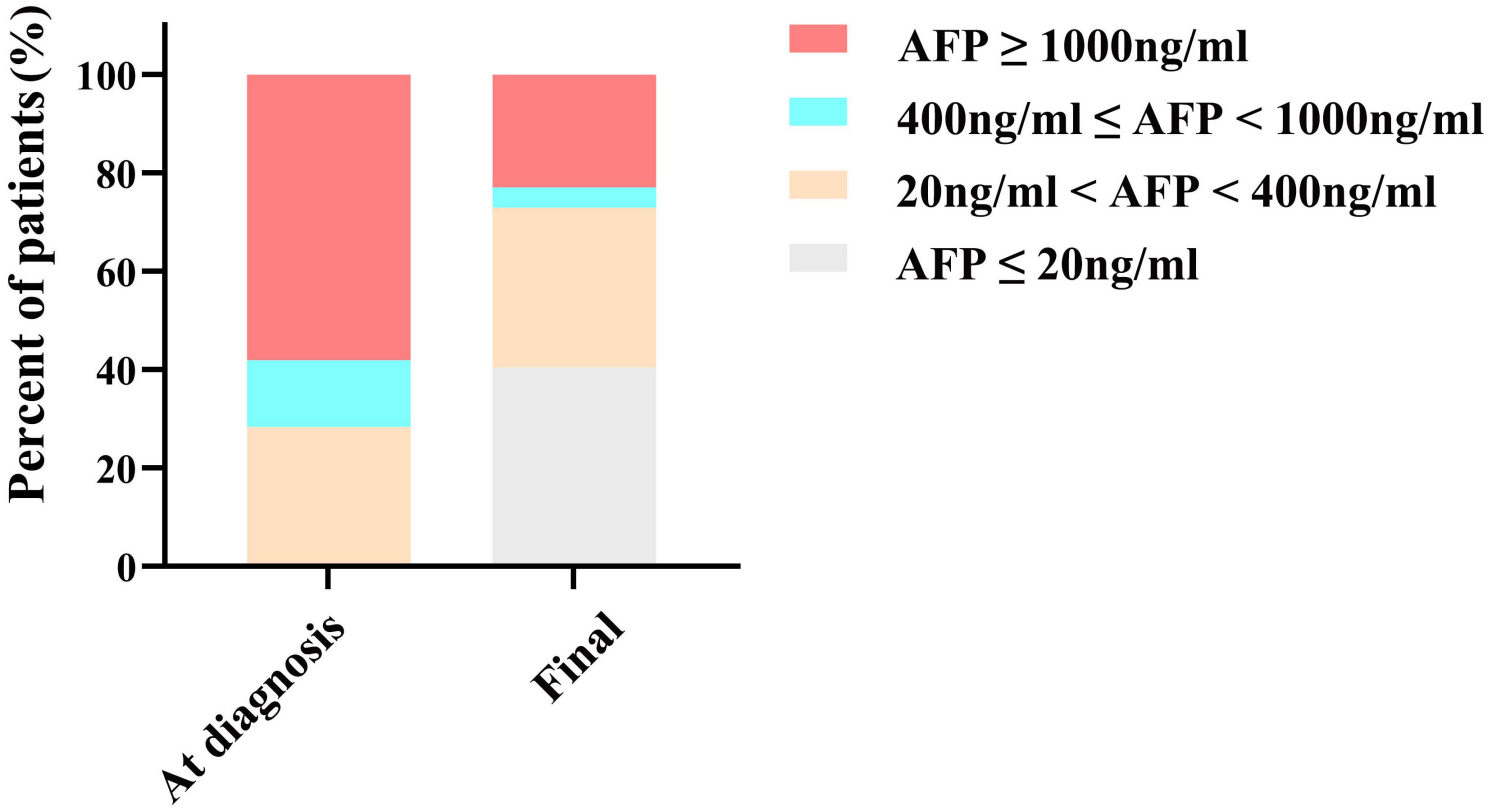
Histogram of AFP status at diagnosis and final. AFP, alpha-fetoprotein.

The median AFP levels at diagnosis and at the final assessment were 1,210 ng/mL (IQR, 292, 8,620) and 44.9 ng/mL (IQR, 7.78, 835), respectively. As depicted in **Figure 2**, at diagnosis, AFP levels for 21 (28.4%) patients ranged from 20 to 400 ng/mL, 10 (13.5%) patients were within the 400 to 1000 ng/mL range, and 43 (58.1%) patients were at or above 1000 ng/mL. Following conversion therapy, there was a notable reduction in final AFP levels: 30 (40.5%) patients had levels of ≤20 ng/mL, 24 (32.4%) patients ranged from 20 to 400 ng/mL, 3 (4.1%) patients were within the 400 to 1000 ng/mL range, and 17 (23.0%) patients were at or above 1000 ng/mL.

The median AFP variation, calculated using the methodological formula, was 94.1% (IQR, 53.2%, 99.2%). The optimal cutoff value of AFP variation determined by Xtile was 80%, dividing patients into AFP responders (n=52, AFP variation ≥80%) and non-responders (n=22, AFP variation <80%). As shown in **Table 1**, clinical characteristics were generally balanced between the two groups, with the exception of AFP level at diagnosis, final AFP levels, and pathological complete response. The AFP responders had higher median AFP levels at diagnosis (1770 ng/mL vs 1210 ng/mL, *P=0.016*), a higher proportion of final AFP levels < 1000ng/mL (88.5% vs 50.0%, *P<0.001*), and a higher rate of pathologic complete response (32.7% vs 4.5%, *P=0.009*).

### Association of AFP response and AFP levels with RFS

**Figure 3:**
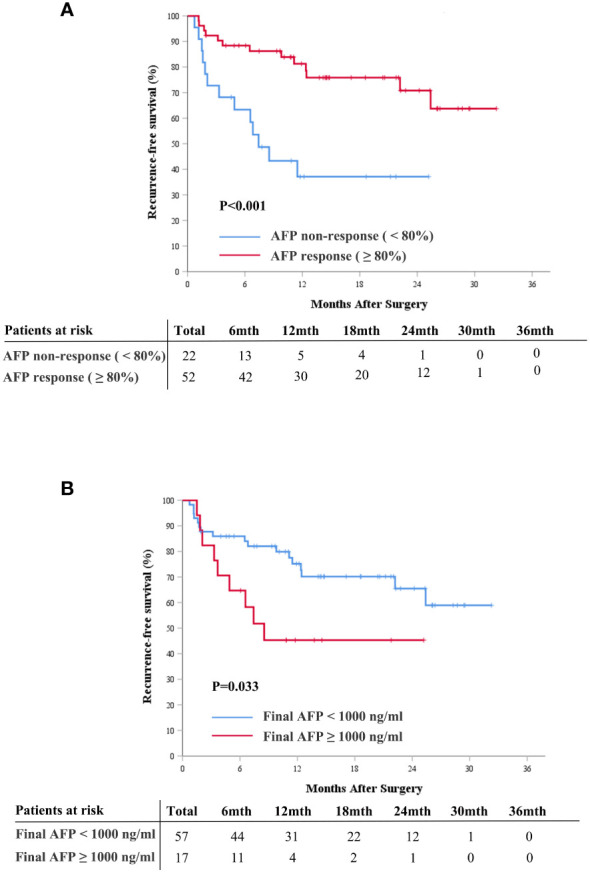
Cumulative recurrence-free survival curves comparison for patients with and without AFP response (≥ 80%) **(A)**, and for patients with final AFP ≥ 1000 ng/mL and < 1000 ng/mL **(B)**. AFP, alpha-fetoprotein.

The median follow-up duration in this study was 14.88 months (IQR, 10.57-24.38). The median RFS for the entire cohort was not reached (95% confidence interval [CI], 22.2, not reached), and the 1- and 2-year RFS rates were 68.0% (95% CI, 57.7%, 80.2%) and 60.0% (95% CI, 48.0%, 75.1%), respectively (**Supplementary Figure S1**). Survival analysis showed that AFP responders had better postoperative RFS than non-responders (*P<0.001*, **Figure 3A**). The median postoperative RFS was not reached in the AFP responder group, with 1-year and 2-year RFS rates of 81.3% (95%CI, 70.8%, 93.2%) and 70.8% (95%CI, 57.1%, 87.8%), respectively. In contrast, the AFP non-responder group had a median postoperative RFS of 7.43 months (95%CI, 4.9, not reached), with 1-year and 2-year RFS rates at 37.1% (95%CI, 20.8%, 66.3%) and 37.1% (95%CI, 20.8%, 66.3%), respectively.

Additionally, we explored the association between AFP levels at diagnosis, final AFP levels, and postoperative RFS. Patients were categorized into high and low AFP groups using previously documented cutoff values of 200 ng/mL, 400 ng/mL, and 1,000 ng/mL. The results revealed that elevated final AFP levels correlated with poor RFS, while AFP levels at diagnosis were not indicative of prognosis (**Figure 3B**, **Supplementary Figures S2, S3**).

Univariable and multivariable Cox proportional hazard regression models were performed to explore the independent preoperative predictive significance of AFP response and AFP levels on postoperative RFS. Univariable analysis revealed that only AFP response (hazard ratio [HR], 0.264; 95% confidence interval [CI], 0.119 to 0.589; *P=0.001*) and final AFP levels (HR, 2.395; 95% CI, 1.047 to 5.477; *P=0.039*) were statistically significant variables (**Table 2**). In the multivariable analysis, after adjustment for other preoperative variables with a P value < 0.1, AFP response remained an independent preoperative predictor for postoperative RFS (HR, 0.332; 95% CI, 0.129 to 0.857; *P=0.023*, **Table 2**).

**Table 2:** Univariable and multivariable analysis of risk factors related to RFS.

Variables	Univariable Analysis		Multivariable Analysis	
	HR (95% CI)	*P Value*	HR (95% CI)	*P Value*
**Age,** ≥65 vs <65, years	0.341 (0.080, 1.447)	0.145		
**Gender,** male vs female	0.759 (0.304, 1.897)	0.555		
**HBsAg,** positive vs negative	4.490 (0.608, 33.173)	0.141		
**Liver cirrhosis,** present vs absent	1.022 (0.463, 2.255)	0.958		
**Combine TACE,** yes vs no	0.477 (0.199, 1.138)	0.095	0.557 (0.208, 1.494)	0.245
**Conversion time,** months	0.833 (0.667, 1.041)	0.108		
**Radiological response,** CR vs PR/SD	0.251 (0.059, 1.063)	0.061	0.336 (0.077, 1.470)	0.148
**Tumor diameter,** cm	0.963 (0.882, 1.050)	0.391		
**Tumor number,** multiple vs solitary	1.425 (0.646, 3.143)	0.381		
**Macrovascular invasion,** present vs absent	0.466 (0.202, 1.075)	0.073	0.545 (0.224, 1.330)	0.183
**AFP level at diagnosis,** ≥1000 vs <1000, ng/ml	0.800 (0.369-1.732)	0.571		
**Final AFP level,** ≥1000 vs <1000, ng/ml	2.395 (1.047, 5.477)	**0.039**	1.147 (0.419, 3.141)	0.789
**AFP response,** ≥80% vs <80%	0.264 (0.119, 0.589)	**0.001**	0.332 (0.129, 0.857)	**0.023**

HBsAg, hepatitis B surface antigen; TACE, transarterial chemoembolization; CR, Complete response; PR, Partial response; SD, Stable disease; AFP, alpha-fetoprotein; HR, hazard ratio; CI, confidence intervals.

Bold indicates statistical significance.

### APF response combined with radiological tumor response

**Figure 4:**
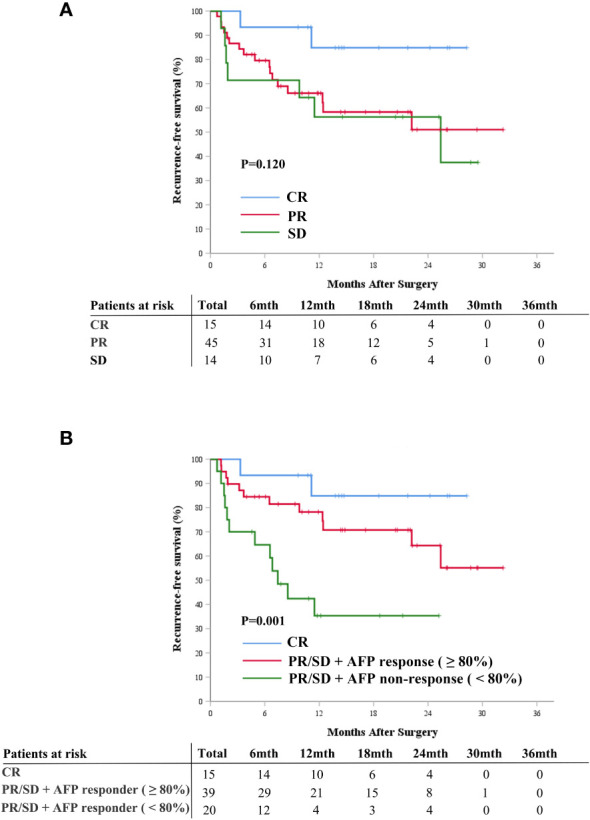
Cumulative recurrence-free survival curves comparison for patients with radiographic responses of CR, PR, and SD **(A)**, and for patients with CR, patients with PR/SD and AFP response, and patients with PR/SD without AFP response **(B)**. AFP, alpha-fetoprotein; CR, complete response; PR, partial response; SD, stable disease.

The Kaplan-Meier curve analysis for radiological response is depicted in **Figure 4A**. Patients who achieved complete response exhibited better postoperative RFS compared to those with partial response or stable disease (**Figure 4A**). Integrating AFP response with radiological response facilitated the further stratification of patients into distinct risk groups: those with complete response experienced the most favorable postoperative RFS, followed by patients with partial response/stable disease and AFP response, whereas patients with partial response/stable disease but lacking AFP response demonstrated the least favorable postoperative RFS (*P=0.001*, **Figure 4B**).

### Sensitivity analysis of AFP response

To mitigate potential heterogeneity in the 80% cutoff value chosen by Xtile, which might be specific to our present cohort, we explored alternative cutoff values of 50% and 20%, as reported in previous literature, for defining AFP responses. We also conducted analyses using these AFP response cutoffs (20%, 50%, and 80%) in a subgroup with initial AFP levels exceeding 200 ng/mL. As demonstrated in **Table 3**, **Supplementary Figures S4, S5**, AFP response consistently correlated with improved postoperative RFS across all examined cutoffs, including in the subgroup with an AFP level greater than 200 ng/mL at diagnosis (all *P<0.05*).

**Table 3:** Sensitivity analysis.

Groups	HR	95%CI	*P value*
AFP > 20ng/ml cohort			
AFP response vs AFP non-response, 50% as the cutoff	0.374	0.166, 0.841	**0.017**
AFP response vs AFP non-response, 20% as the cutoff	0.423	0.185, 0.965	**0.041**
AFP≥200ng/ml cohort			
AFP response vs AFP non-response, 80% as the cutoff	0.183	0.073, 0.462	**<0.001**
AFP response vs AFP non-response, 50% as the cutoff	0.277	0.109, 0.702	**0.007**
AFP response vs AFP non-response, 20% as the cutoff	0.324	0.126, 0.835	**0.020**

AFP, alpha-fetoprotein; HR, hazard ratio; CI, confidence intervals.

Bold indicates statistical significance.

## Discussion

In this multicenter retrospective study, we systematically assessed the association between serum AFP level variations and postoperative RFS of patients with initially unresectable HCC who undergoing salvage hepatectomy after conversion therapy regimens based on TKIs combined with α-PD-1. Our findings highlight that a reduction of AFP by ≥80% following conversion therapy is closely linked with improved postoperative RFS in patients with baseline AFP levels above 20 ng/mL. This “20-80” rule enriches the clinical decision-making framework for salvage hepatectomy following TKI and α-PD-1 based conversion therapy, particularly in light of the procedure’s invasive nature. It potentially aids clinicians and patients in evaluating the efficacy of preoperative therapy and in making informed choices about proceeding with surgery.

AFP, recognized as a key biomarker in HCC management, has shown renewed significance in predicting treatment outcomes through its dynamic variations, commonly referred to as the “AFP response” (19, 23, 24, 30, 31). This concept gained attention with two pivotal studies in 2009, published in the Journal of Clinical Oncology, which demonstrated the utility of AFP response, using thresholds of 50% and 20%, as a marker post-locoregional and systemic chemotherapy for HCC, respectively. Our study, however, adopts a higher cutoff value of 80% for AFP response. This decision aligns with the distinct characteristics of our patient cohort, who underwent salvage hepatectomy following successful conversion therapy. The choice of a higher cutoff is justified by our cohort’s median AFP level at diagnosis, which was notably high at 1,210 ng/mL and then significantly reduced to 44.9 ng/mL before hepatectomy. Furthermore, our selection of this threshold is based on the statistical rationale provided by Xtile, offering a more methodologically robust alternative to the arbitrary 50% or 20% cutoffs employed in previous studies (28). In our context, the 80% cutoff offers the advantage of accurately reflecting true AFP changes attributable to HCC, while reducing the influence of varying liver disease conditions and inter-laboratory discrepancies.

Radiological evaluation for salvage hepatectomy is a routine clinical practice for assessing potential treatment efficacy. Radiological assessments present specific limitations, including subjectivity, interobserver heterogeneity, and challenges arising from cirrhosis, post-treatment scarring, vascular occlusion, and even contrast agent injection timing (23, 32). A primary concern with radiological assessment is its inability to accurately predict prognosis (33, 34). However, our findings show that patients who achieve complete radiological response have better prognoses than those with partial response or stable disease, suggesting that preoperative imaging continues to be a reliable predictor for salvage hepatectomy prognosis. Additionally, our results suggest that integrating AFP response with preoperative radiological tumor response could offer potential advantages for prognostic evaluation. Radiological tumor response contributes to the assessment of tumor resectability and plays a vital role in devising comprehensive surgical resection strategies. However, it represents only macroscopic tumor reduction, not microscopic liver metastases, which are often the source of postoperative recurrence. Conversely, AFP response may reflect the overall subclinical disease burden and provide further information on the overall improvement in tumor burden. As a result, the potential benefits of combining AFP response with radiological tumor response in the salvage hepatectomy population merit further investigation.

The present study is noteworthy for several reasons. Firstly, to our knowledge, this is the first study assessing AFP response as a preoperative surrogate marker for RFS after salvage hepatectomy. Secondly, a critical advantage of utilizing AFP response in our study is its role in providing preoperative guidance. This application is distinct from its conventional use in assessing the effectiveness of systemic or local treatments for HCC, which is typically evaluated one month post-treatment initiation (19–21). Our approach advances the understanding of AFP response, offering valuable insights prior to the commencement of salvage hepatectomy, thereby enhancing the decision-making process in clinical settings. Thirdly, the findings of our study have significant implications for neoadjuvant therapy in HCC. While clinical trials in this area are extensive, benefiting from the integration of novel systemic antitumor agents, there remains a pressing need for an effective, straightforward preoperative efficacy evaluation indicator. Our findings may illuminate a novel direction suggesting that attention may be warranted toward the AFP response in future clinical trials of neoadjuvant therapy for HCC, with the aim to ascertain its capacity to serve as an efficacious marker of the oncological advantage derived from neoadjuvant therapy.

Our study has several limitations. Firstly, its retrospective nature introduces the possibility of selection bias. Secondly, the limited sample size of our study posed constraints on the statistical power. Consequently, there is a pressing need for larger, multi-center prospective studies to confirm the correlation between AFP response and postoperative prognosis in salvage hepatectomy and to establish a standardized AFP response cutoff. Thirdly, the relatively short follow-up duration of our study limits our ability to assess the association between AFP response and postoperative overall survival. This limitation highlights the necessity for future research to more comprehensively explore the relationship between AFP response and overall survival, given the critical importance of long-term survival outcomes in evaluating the effectiveness of salvage hepatectomy. Fourthly, our study focused exclusively on AFP without including other relevant tumor markers, such as protein induced by vitamin K absence or antagonist-II. Lastly, limited by the retrospective non-interventional nature of our study, we exclusively included patients who underwent salvage hepatectomy, lacking a comparable cohort that met resection criteria but did not proceed with salvage hepatectomy, largely due to the inherent difficulties in data collection. Consequently, within the confines of our investigation, we could not definitively conclude whether salvage hepatectomy yields the greater oncologic advantage over non-surgical treatments, such as maintaining the original systemic therapeutic regimen, in patients exhibiting an AFP response.

In summary, our study highlights that AFP response may serve as a simple, reproducible, and reliable preoperative biological surrogate marker for postoperative RFS in patients undergoing salvage hepatectomy. The “20-80” rule based on AFP response could be helpful for clinicians to preoperatively stratify the risk of patients undergoing salvage hepatectomy, enabling identification and management of those unlikely to benefit from this procedure.

## Data availability statement

The original contributions presented in the study are included in the article/**Supplementary Material**. Further inquiries can be directed to the corresponding authors.

## Ethics statement

All procedures performed in studies involving human participants were by the ethical standards of the institutional and/or national research committee. And this research was conducted ethically by the World Medical Association Declaration of Helsinki.

## Author contributions

K-YL: Conceptualization, Data curation, Formal Analysis, Investigation, Methodology, Resources, Software, Supervision, Visualization, Writing – original draft, Writing – review & editing. J-XZ: Conceptualization, Data curation, Formal Analysis, Funding acquisition, Investigation, Methodology, Supervision, Writing – original draft. Z-WL: Conceptualization, Data curation, Formal Analysis, Investigation, Methodology, Project administration, Supervision, Writing – original draft. Q-JC: Conceptualization, Data curation, Investigation, Methodology, Project administration, Resources, Software, Supervision, Writing – original draft. L-PL: Data curation, Investigation, Methodology, Project administration, Resources, Writing – original draft. J-HC: Investigation, Methodology, Project administration, Resources, Software, Supervision, Writing – original draft. KuW: Conceptualization, Data curation, Validation, Writing – review & editing. ST: Data curation, Validation, Writing – review & editing. Z-BZ: Data curation, Writing – review & editing. S-FW: Data curation, Methodology, Writing – review & editing. J-DL: Data curation, Formal Analysis, Supervision, Writing – review & editing. KaW: Data curation, Writing – review & editing. LZ: Data curation, Writing – review & editing. S-MZ: Data curation, Writing – review & editing. M-MW: Data curation, Writing – review & editing. K-CL: Data curation, Writing – review & editing. TY: Writing – review & editing. Y-YZ: Conceptualization, Data curation, Formal Analysis, Investigation, Methodology, Project administration, Software, Supervision, Validation, Writing – review & editing.
