# Supplementary material for: Serum alpha-fetoprotein response as a preoperative prognostic indicator in unresectable hepatocellular carcinoma with salvage hepatectomy following conversion therapy: a multicenter retrospective study
Source: Front Immunol. 2024 Feb 16;15:1308543. doi: 10.3389/fimmu.2024.1308543 (PMC10904489; doi:10.3389/fimmu.2024.1308543)
Supplement: Supplementary file 1 [file DataSheet_1.docx]

**Serum Alpha-fetoprotein Response as a Preoperative Prognostic Indicator in Unresectable Hepatocellular Carcinoma with Salvage Hepatectomy Following Conversion Therapy: A Multicenter Retrospective Study**

**Contents**

[Figure S1. Cumulative recurrence-free survival curve of patients undergoing salvage liver resection for HCC after conversion therapy with TKIs and α-PD-1-based Regimen. 2](#_Toc136288467)

[Figure S2. Cumulative recurrence-free survival curve comparison for patients with final AFP ≥ 200 ng/ml and < 200 ng/ml (A), and for patients with final AFP ≥ 400 ng/ml and < 400 ng/ml (B). 3](#_Toc136288468)

[Figure S3. Cumulative recurrence-free survival curve comparison for patients with AFP ≥ 200 ng/ml and < 200 ng/ml at diagnosis (A), for patients with AFP ≥ 400 ng/ml and < 400 ng/ml at diagnosis (B), and for patients with AFP ≥ 1000 ng/ml and < 1000 ng/ml at diagnosis (C). 4](#_Toc136288469)

[Figure S4. Cumulative recurrence-free survival curves comparison for patients with and without AFP response, using previously reported cut-off values of 50% (A) and 20% (B). 5](#_Toc136288470)

[Figure S5. Cumulative recurrence-free survival curves comparison for patients with AFP ≥200 ng/ml at diagnosis with and without AFP response using different cutoffs of 80% (A), 50% (B), and 20% (C). 6](#_Toc136288471)

# Figure S1. Cumulative recurrence-free survival curve of patients undergoing salvage liver resection for HCC after conversion therapy with TKIs and α-PD-1-based Regimen.


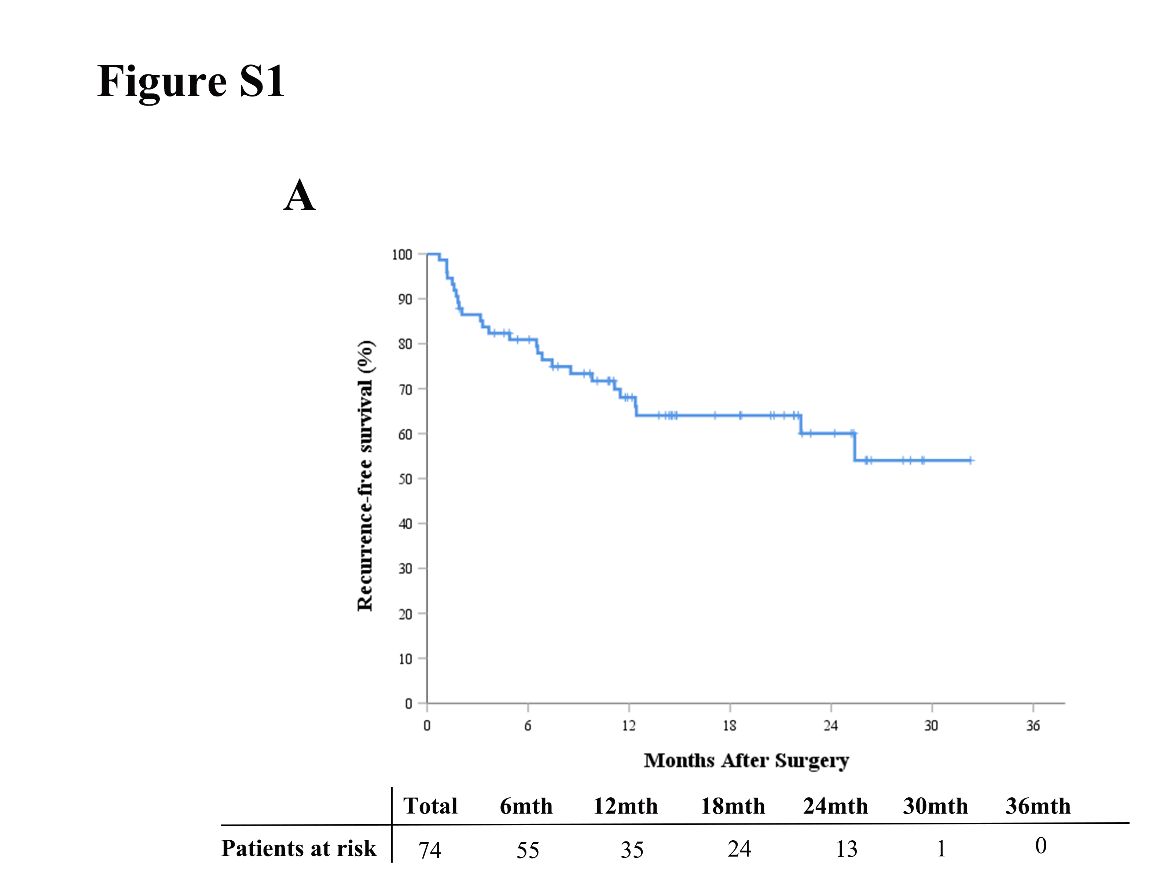


Abbreviation: TKI, tyrosine Kinase Inhibitor; PD-1, programmed death 1.

# Figure S2. Cumulative recurrence-free survival curve comparison for patients with final AFP ≥ 200 ng/ml and < 200 ng/ml (A), and for patients with final AFP ≥ 400 ng/ml and < 400 ng/ml (B).


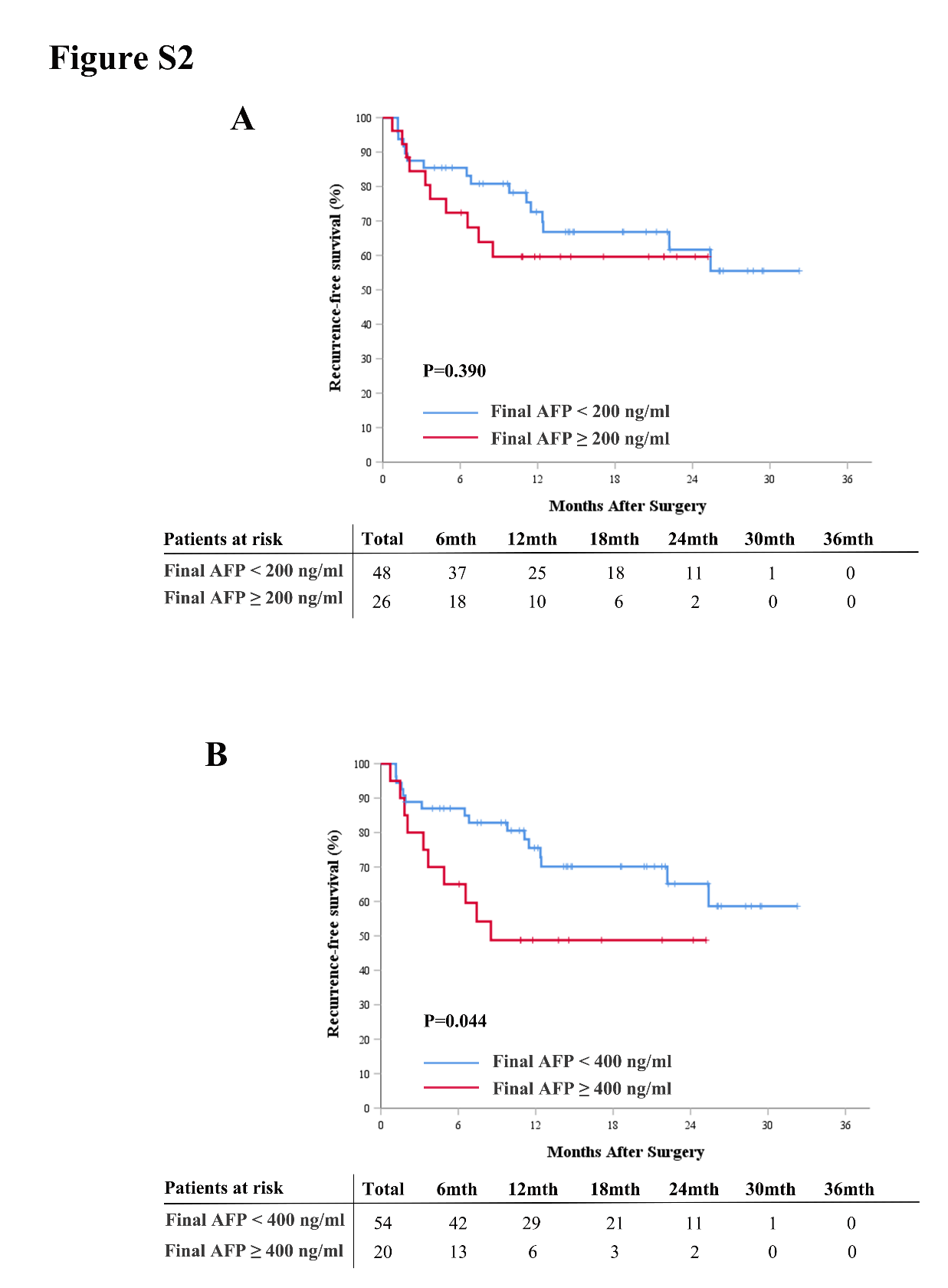


Abbreviation: AFP, alpha-fetoprotein.

# Figure S3. Cumulative recurrence-free survival curve comparison for patients with AFP ≥ 200 ng/ml and < 200 ng/ml at diagnosis (A), for patients with AFP ≥ 400 ng/ml and < 400 ng/ml at diagnosis (B), and for patients with AFP ≥ 1000 ng/ml and < 1000 ng/ml at diagnosis (C).

**
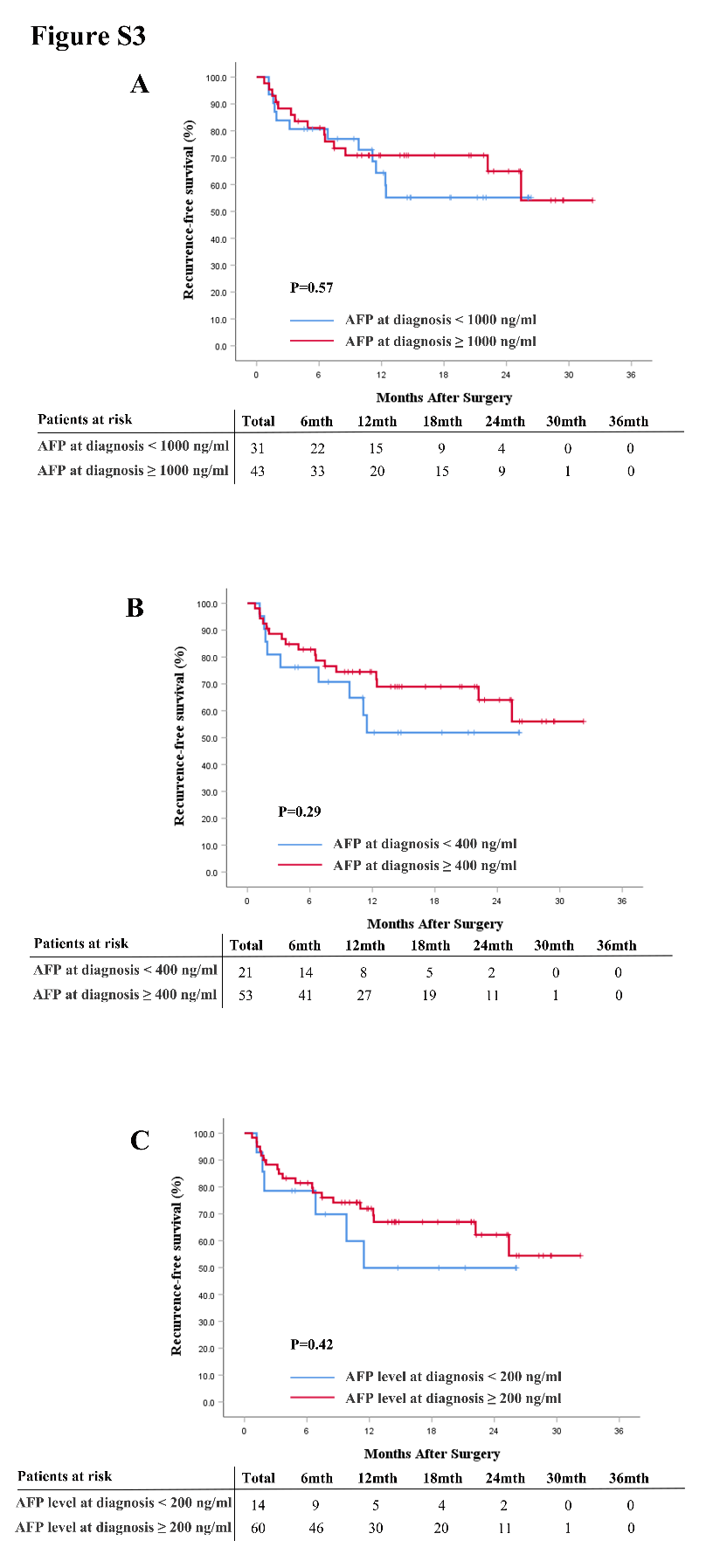
**

Abbreviation: AFP, alpha-fetoprotein.

# Figure S4. Cumulative recurrence-free survival curves comparison for patients with and without AFP response, using previously reported cut-off values of 50% (A) and 20% (B).


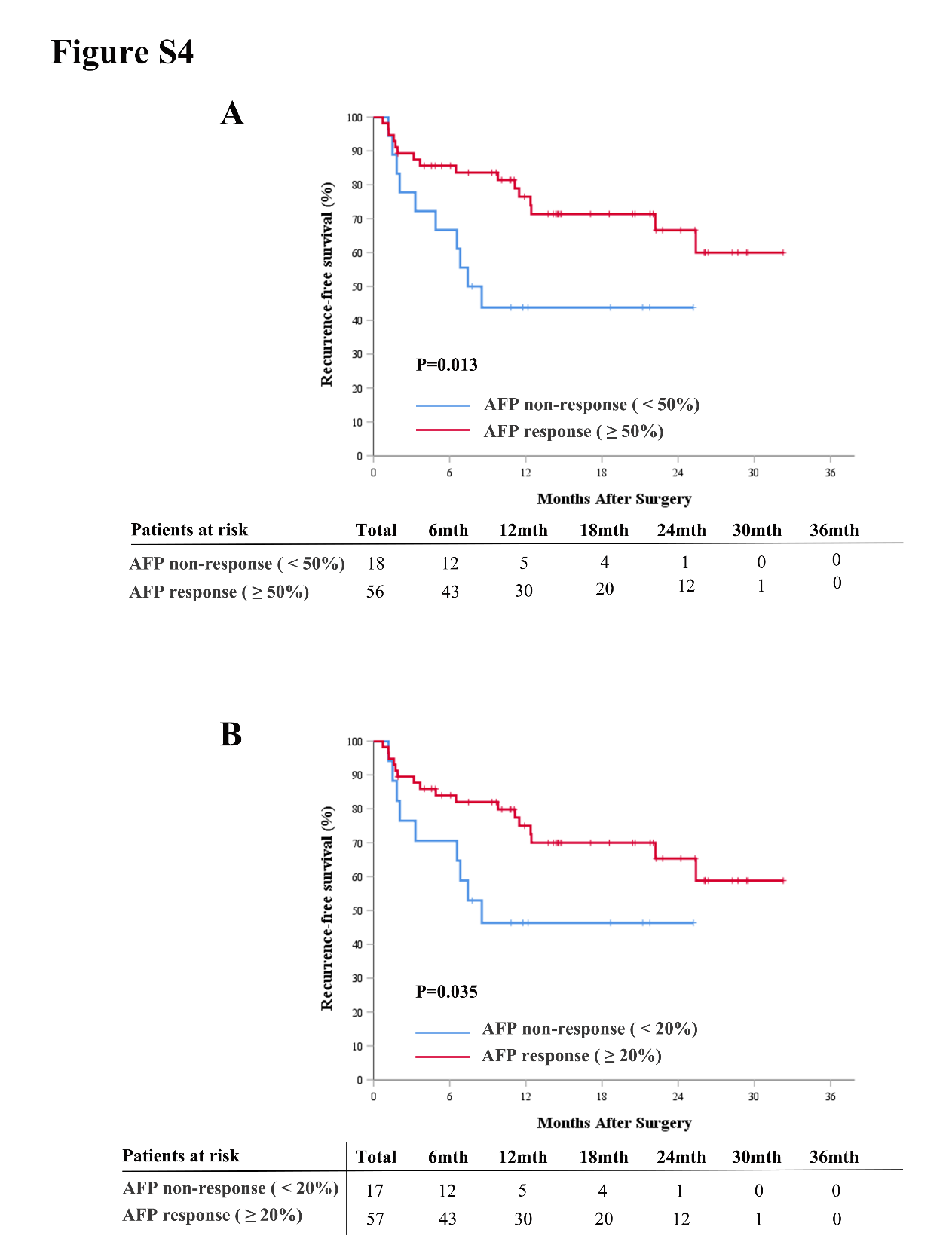


Abbreviation: AFP, alpha-fetoprotein.

# Figure S5. Cumulative recurrence-free survival curves comparison for patients with AFP ≥200 ng/ml at diagnosis with and without AFP response using different cutoffs of 80% (A), 50% (B), and 20% (C).


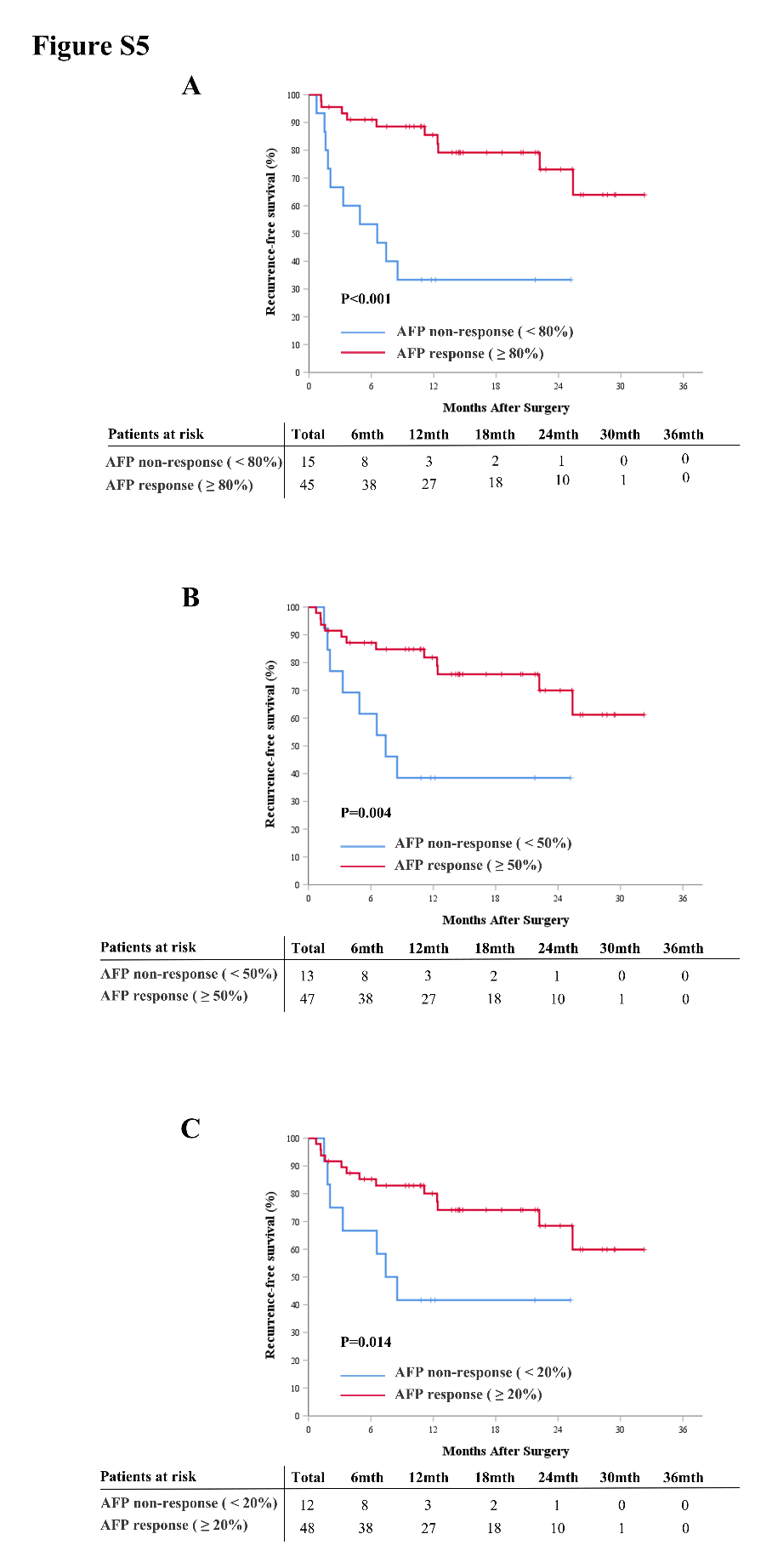


Abbreviation: AFP, alpha-fetoprotein.
